# Complete Genome Sequence of the Type Strain *Corynebacterium testudinoris* DSM 44614, Recovered from Necrotic Lesions in the Mouth of a Tortoise

**DOI:** 10.1128/genomeA.00784-15

**Published:** 2015-07-30

**Authors:** Christian Rückert, Martin Kriete, Sebastian Jaenicke, Anika Winkler, Andreas Tauch

**Affiliations:** a Department of Biology, Massachusetts Institute of Technology (MIT), Cambridge, Massachusetts, USA; b Institut für Genomforschung und Systembiologie, Centrum für Biotechnologie (CeBiTec), Universität Bielefeld, Bielefeld, Germany; c Bioinformatics Resource Facility (BRF), Centrum für Biotechnologie (CeBiTec), Universität Bielefeld, Bielefeld, Germany

## Abstract

The complete genome sequence of the type strain *Corynebacterium testudinoris* DSM 44614 from the mouth of a tortoise comprises 2,721,226 bp with a mean G+C content of 63.14%. The automatic annotation of the genome sequence revealed 4 rRNA operons, 51 tRNA genes, 7 other RNA genes, and 2,561 protein-coding regions.

## GENOME ANNOUNCEMENT

The taxonomic characterization of the species *Corynebacterium testudinoris* was published in 2001 and was based on microbiological, biochemical, and molecular genetic data of the single isolate M935/96/4^T^ (1). This type strain, deposited in the German culture collection DSMZ as DSM 44614, was initially isolated from necrotic lesions in the mouth of a tortoise and in mixed culture with *Escherichia coli*, a *Pseudomonas* species, and a *Streptococcus* species (1). Moreover, thirteen corynebacterial strains with very similar biochemical and physiological characteristics were isolated from young pigs suffering from respiratory infections (2). These isolates were biochemically homogeneous and displayed 99.8 to 99.9% nucleotide sequence similarity to the 16S rRNA gene of *C. testudinoris* DSM 44614 (2). *C. testudinoris* strains were moreover isolated from a fish processing wastewater treatment plant in India (GenBank accession number KC161906) and from semen of a silver barb, *Barbodes gonionotus*, in Thailand (GenBank accession number KF699880). These data suggest that *C. testudinoris* represents a commensal bacterium in a broad spectrum of animals. In this study, we determined the complete genome sequence of the type strain *C. testudinoris* DSM 44614 to provide a solid basis for further molecular genetic analyses of this corynebacterium.

Purified genomic DNA of *C. testudinoris* DSM 44614 was obtained from the Leibniz Institute DSMZ (Braunschweig, Germany). A whole-genome shotgun library was constructed with the Nextera DNA sample preparation kit (Illumina) and was sequenced in a paired-end run using the MiSeq reagent kit v2 (500 cycles) and the MiSeq desktop sequencer (Illumina). This shotgun-sequencing approach yielded 2,720,454 paired reads and 481,239,106 detected bases. An assembly of the paired reads was performed with the Roche GS De Novo Assembler software (Newbler, release 2.8) and resulted in 27 scaffolds including 33 scaffolded contigs. For further scaffolding and gap closure, an additional 7-kb mate pair library was prepared with the Nextera mate pair sample preparation kit according to the gel-plus protocol. This DNA library was sequenced with the MiSeq reagent kit v3 (600 cycles), yielding 647,514 mate pair reads that were added to the initial Newbler assembly. The gap closure step of this genome project was supported by the Consed software package (version 26) (3). The regional gene prediction in the complete genome sequence of *C. testudinoris* DSM 44614 was performed with the Prodigal software (4) and the functional annotation of the detected protein-coding regions was carried out by the IMG/ER pipeline (5). Regional and functional genome data were finally visualized with the GenDB software (6).

The genome of *C. testudinoris* DSM 44614 consists of a circular chromosome with a size of 2,721,226 bp and a mean G+C content of 63.14%. The automatic annotation of the genome sequence revealed 4 rRNA operons, 51 tRNA genes, 7 other RNA genes, and 2,561 protein-coding regions, including 2,081 protein-coding genes with functional predictions (5). The functional annotation of the *C. testudinoris* DSM 44614 genome includes 666 genes coding for transmembrane proteins and 100 genes encoding protein precursors with signal peptides. The coding density of the *C. testudinoris* DSM 44614 genome sequence is 91.62% (5).

### Nucleotide sequence accession number.

This genome project has been deposited in the GenBank database under the accession no. CP011545.
